# Supplementary material for: Integration and evaluation of a gradient-based needle navigation system for percutaneous MR-guided interventions
Source: PLoS One. 2020 Jul 24;15(7):e0236295. doi: 10.1371/journal.pone.0236295 (PMC7380643; doi:10.1371/journal.pone.0236295)
Supplement: Attachment — Submitted filename: Response_to_Reviewers.docx [file pone.0236295.s002.docx]

Hannover Medical School, Carl-Neuberg Street 1, 30625 Hannover

**Zhongliang Zu, Ph.D.**

**Academic Editor**

**PLOS ONE**

**Department of Diagnostic and Interventional Radiology**

**Prof. Dr. Frank Wacker, Director**

OE 8220

Dr. Bennet Hensen

Phone: 0511 532-2690

Hensen.Bennet@mh-hannover.de

Hannover Medical School

Carl-Neuberg Street 1

30625 Hannover

www.mh-hannover.de

**Submission of our original research paper „Integration and Evaluation of a Gradient-based Needle Navigation System for Percutaneous MR-guided Interventions“**

08/06/2020

Dear Dr. Zu,

I would like to thank you and your reviewers for the time and effort you have put into the review of our manuscript. We really appreciate all of your input. The following is a detailed summary of the replies and changes made in our manuscript in direct response to each specific reviewer comment.

Sincerely,

Bennet Hensen and Frank Wacker, for all authors

***Journal requirements:***

1. Please ensure that your manuscript meets PLOS ONE's style requirements, including those for file naming. The PLOS ONE style templates can be found at http://www.plosone.org/attachments/PLOSOne_formatting_sample_main_body.pdf and http://www.plosone.org/attachments/PLOSOne_formatting_sample_title_authors_affiliations.pdf

**The manuscript style was adapted to the style requirements.**

2. Please include the following information in your methods section:

•	Please specify the source from which animals were obtained

•	Please provide details of animal welfare and housing (e.g., shelter, food, water, environmental enrichment)

•	Please complete and submit a copy of the ARRIVE Guidelines checklist, a document that aims to improve experimental reporting and reproducibility of animal studies for purposes of post-publication data analysis and reproducibility: https://www.nc3rs.org.uk/arrive-guidelines. Please include your completed checklist as a Supporting Information file. Note that if your paper is accepted for publication, this checklist will be published as part of your article.

**Detailed information on animal source and animal welfare and housing was added.**

**The ARRIVE Guidelines checklist was uploaded.**

3. Thank you for stating the following in the Competing Interests section:

(…)

**An updated Funding Statement and a Competing Interests Statement was included in the cover letter.**

4. PLOS requires an ORCID iD for the corresponding author in Editorial Manager on papers submitted after December 6th, 2016.

**The ORCID iD was added.**

5. Please upload a new copy of Figure 4 as the detail is not clear. Please follow the link for more information: https://blogs.plos.org/plos/2019/06/looking-good-tips-for-creating-your-plos-figures-graphics/

**All Figures are now replaced with high DPI versions. Figure 4 has been adjusted and homogenized, the sequence diagrams now look the same.**

***Review Comments to the Author:***

***Reviewer #1***: This is an interesting paper. The following points should be considered to further improve it.

1. Figures 1, 3, 4 are very low resolution. Please replace with high DPI version.

**All Figures are now replaced with high DPI versions.**

2. The results describe the performance of the proposed system, but no control group was presented. The Authors should at least provide a comparison based on the literature (e.g. needle insertion time, error in plane and in 3D for competitor solutions).

**We included a comparison based on the literature in the “Discussion” section (line 533 – 536).**

3. The Authors write on Page 21 "The multi-slice real-time images were acquired and displayed in a mosaic mode to enable device guidance while allowing the operator to adapt to both organ movement due to breathing and tissue deformation/displacement due to needle insertion."

Later however the Authors write (on Page 23) "we targeted only organs that have minimal motion during breathing". Please explain the reason for this choice, since the system should be able to cope with this, right?

**The current system includes static as well as real time information. This is now explained in the “Discussion” section (line 587 – 592).**

4. On page 23 the Authors write "we observed overshooting of the needle in both, phantom and animal experiments". A relatively simple solution to this problem would be a depth gauge on the needle. Is this not possible with the proposed system?

**We agree with the reviewer that a depth gauge could be a simple solution. However, elasticity of soft tissue in humans, animals and most phantoms cause pushback once the target is reached. In order to adjust for elastic resistance, overshooting is somewhat intended but the extent is sometimes difficult to foresee, especially when relying on needle tip visualization in static rather than realtime images.**

***Reviewer #2***: The manuscript is interesting and well written. I can see the interest of the system but do the results really support that this helps the IR? not sure (8mm deviation, 4 cases of overshooting)... it is not an issue as it is work in progress, but this should be mentioned in the discussion especially Given the conflict of interests of the authors,

**We agree with the reviewer. This is a prototype system not ready for clinical use. We clarified this in the “Discussion” section (line 613 – 617).**

A couple of points that need to be addressed/corrected to my point of view:

- is the needle holder sterilizable?

**Yes, this information were added in the manuscript (line 113)**

- please provide the characteristics of the BEAT-IRTTT sequences

**Details for BEAT-IRTTT were added in the methods section (line 161 - 164)**

- line 168: in which plane were HASTE images acquired? What was the number of HASTE images?

**The HASTE sequence was acquired in an axial, coronal and transversal plane (line 187).**

- fig7 is mentioned before fig6 in the text

**This was corrected.**

- line 223: what were the reasons to use a T1 or a T2 sequence? please be more precise

**T1 images were selected. This information has been corrected in the manuscript.**

- what were the targets in the pigs? Kidney is rather vague. Same for cervical spine or shoulder. a figure could be helpful

**Spine: the cervical intervertebral foramen to simulate a transforaminal epidural injection**

**Shoulder joint: the anterior edge of the glenoid to simulate an anterior approach to shoulder arthrography**

**Kidney: minor calyx of the lower pole to simulate a nephrostomy**

**Details are now given in the manuscript (line 298-300)**

- results section and measures: given the low spatial resolution of MRI guidance, giving a hundredth of millimeter does not make sense. there is a huge place for errors there. Who made the measurements? how many times were they made?

**We fully agree with the reviewer, that this does not make sense. Therefore, the numbers were rounded.**

- line 282 to 295: you give a lot of measures but the terms are unclear. It would be better to specify clearly which results apply to expriement1/2 and IR1/2

**In the “Results” section, the measurements are given for experiment 1 and 2 and for IR 1 and 2 respectively. We clarified this by adding some information regarding IR 1 and 2.**

- was overshooting recognized at the time of puncture? if not, i believe that statistic should be presented including the outlier target.

**Overshooting was recognized during the intervention by the less experienced radiologist who overestimated elasticity and prematurely finished the respective puncture. For full disclosure the experiments are mentioned but, give the operator error, are not included in the results.**

- line 291: learning curve: how do you compare the learning curve? it was not specified in the method section. Moreover, base on the length of procedure, it seems to be a difference

**An operator not familiar with a guidance system usually improves after the first set of experiments. In order to account for a potential learning curve, we therefore compared experiment 1 and 2 for both IR1 and 2. This was clarified in the “Results” section.**

- please provide a p-value if there is one to compare precision and time to insert the needle between IR1 and IR2

**This information is given in the “Results” section (time to insert: line 463; precision: line 456).**

- line 299 to 305: these are not results. It is a subjective description, please remove

**We agree. The paragraph is omitted.**
